# Supplementary material for: Traits of Bathy Phytochromes and Application to Bacterial Optogenetics
Source: ACS Synth Biol. 2025 Jul 11;14(8):3207–18. doi: 10.1021/acssynbio.5c00337 (PMC12362586; doi:10.1021/acssynbio.5c00337)
Supplement: Supplementary file 1 [file sb5c00337_si_001.pdf]

Traits of bathy phytochromes and application to bacterial optogenetics

Cornelia Böhm[1,2], Kimmo Lehtinen[1], Elina Multamäki[3], Roosa Vanhatalo[1], Oscar Brander[3], Stefanie S. M. Meier[4], Jessica Rumfeldt[1], Andreas Möglich[4], Heikki Takala[1,3]

AFFILIATIONS

[1] Department of Biological and Environmental Science, University of Jyväskylä, Finland

[2] Institute of Biochemistry, Graz University of Technology, Austria

[3] Department of Anatomy, University of Helsinki, Finland

[4] Department of Biochemistry, University of Bayreuth, Germany

*H.T. heikki.p.takala@jyu.fi, +358 46 923 6211

SUPPORTING INFORMATION

Figure S1	S2
Figure S2	S4
Figure S3	S5
Figure S4	S6
Figure S5	S7
Figure S6	S9
Figure S7	S10
Figure S8	S12
Figure S9	S13
Figure S10	S15
Table S1	S17
Table S2	S18
Table S3	S19
Table S4	S20

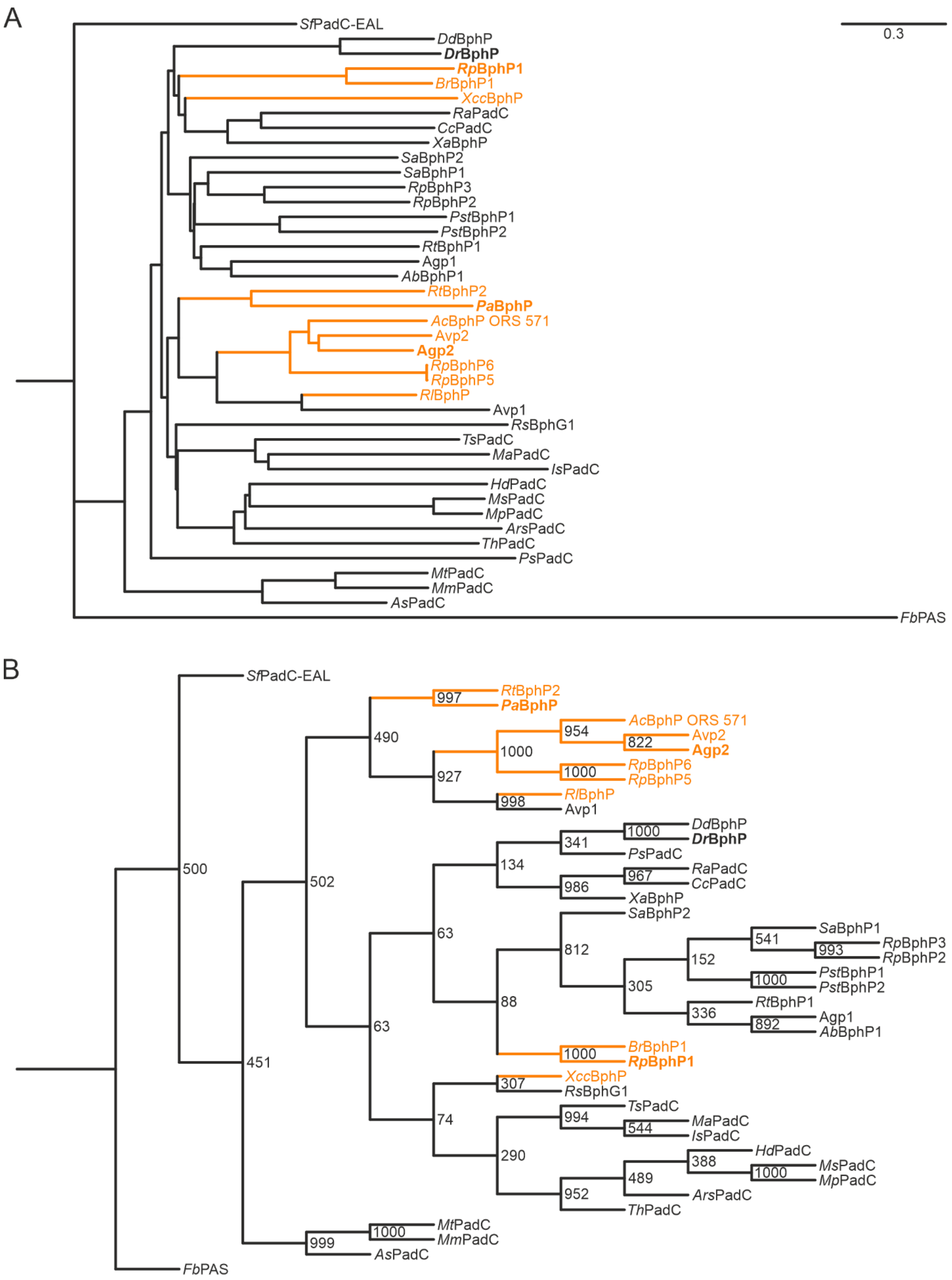
A
SfPadC-EAL
0.3
DdBphP
DrBphP
RpBphP1
BrBphP1
XccBphP
RaPadC
CcPadC
XaBphP
SaBphP2
SaBphP1
RpBphP3
RpBphP2
PstBphP1
PstBphP2
RtBphP1
Agp1
AbBphP1
RtBphP2
PaBphP
AcBphP ORS 571
Avp2
Agp2
RpBphP6
RpBphP5
RlBphP
Avp1
RsBphG1
TsPadC
MaPadC
IsPadC
HdPadC
MsPadC
MpPadC
ArsPadC
ThPadC
PsPadC
MtPadC
MmPadC
AsPadC
FbPAS
B
SfPadC-EAL
997
RtBphP2
PaBphP
490
954
AcBphP ORS 571
1000
822
Avp2
Agp2
927
1000
RpBphP6
RpBphP5
998
RlBphP
Avp1
1000
DdBphP
341
DrBphP
134
PsPadC
967
RaPadC
986
CcPadC
XaBphP
500
502
SaBphP2
SaBphP1
541
RpBphP3
993
RpBphP2
63
812
152
1000
PstBphP1
PstBphP2
305
RtBphP1
336
Agp1
892
AbBphP1
88
63
1000
BrBphP1
RpBphP1
451
307
XccBphP
RsBphG1
TsPadC
994
MaPadC
544
IsPadC
74
HdPadC
388
MsPadC
1000
MpPadC
290
489
ArsPadC
952
ThPadC
1000
MtPadC
MmPadC
999
AsPadC
FbPAS

Figure S1: ***Phylogenetic trees of BphP PSM sequences with known spectral properties,*** *distance* ***(A)*** *vs. bootstrap tree* ***(B)****. BphP sequences were aligned with ClustalO default settings* [1] *(alignment available as supporting file, “Supp_known_aligned.phy”) and cut off after the PHY domain (last residue corresponds to DrBphP T499, before GALGERL motif). Trees were created using the PHYLIP 3.6 package (PROTDIST, FITCH)* [2]*. For the bootstrap tree, 1000 Jackknife subalignments were created using SEQBOOT and distance trees of subalignments were merged with CONSENSE. Trees were visualised with Figtree* [3]*. A PAS domain-containing protein from Flavobacterium bernardetii (FbPAS, NCBI protein ID WP_166125522) serves as outgroup. Known bathy BphPs are represented in orange. Low bootstrap values at some branching points highlight the complicated relationships across BphPs.*

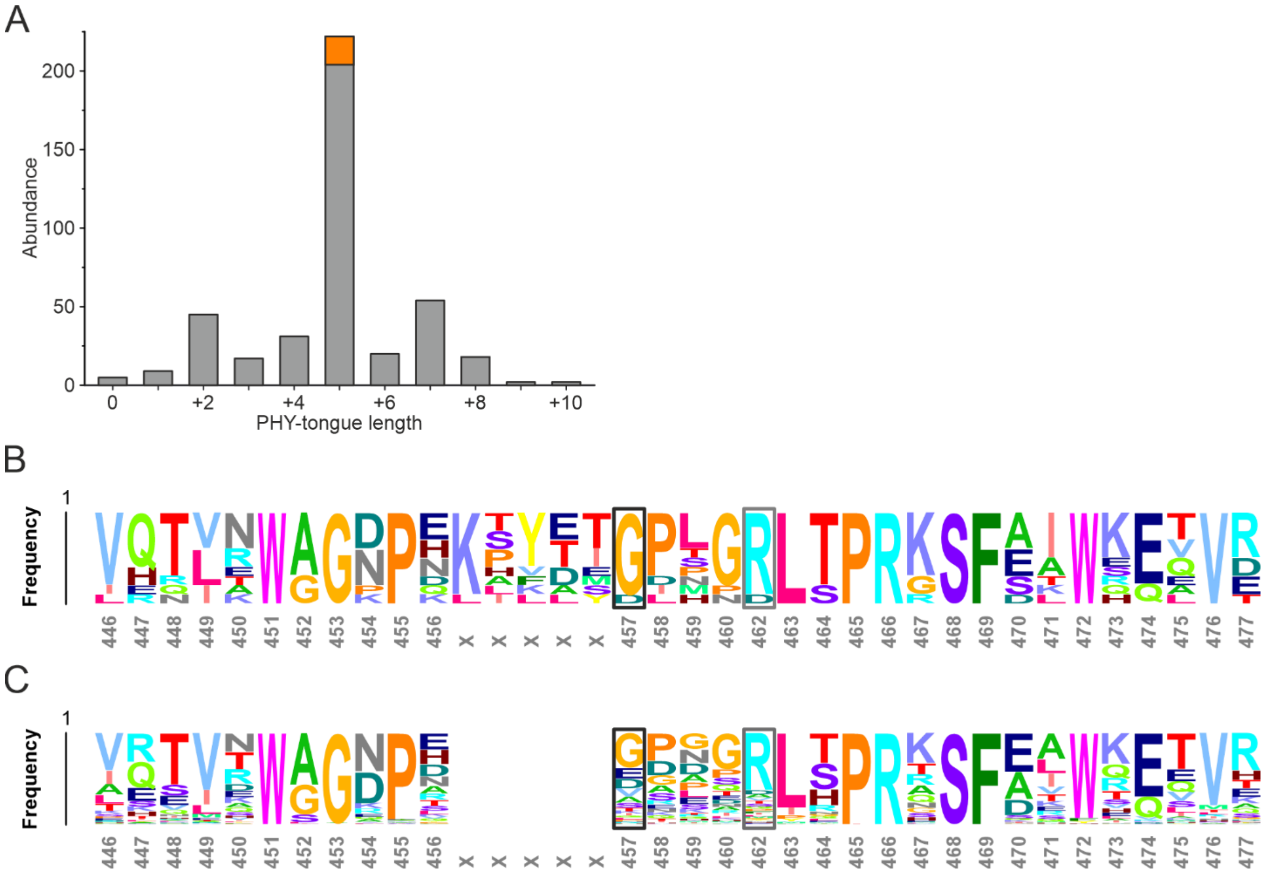

Figure S2: ***PHY-tongue differences across BphPs. (A)*** *Absolute abundance of PHY-tongue lengths found in a ClustalO-aligned compilation of 404 BphP sequences. The tongue length is counted from WAG–5 to PRXSF+8 and given relative to DrBphP (32 residues). Tongue length of known bathy BphPs (+5) is marked in orange.* ***(B-C)*** *kpLogo* [4] *of 11 known bathy BphPs (B) vs. 404 BphP sequences (C). Numbering corresponds to DrBphP, the gap represents differences in tongue length. The glycine residue corresponding to DrBphP D457 is notably more conserved in bathy BphPs than in BphPs overall.*

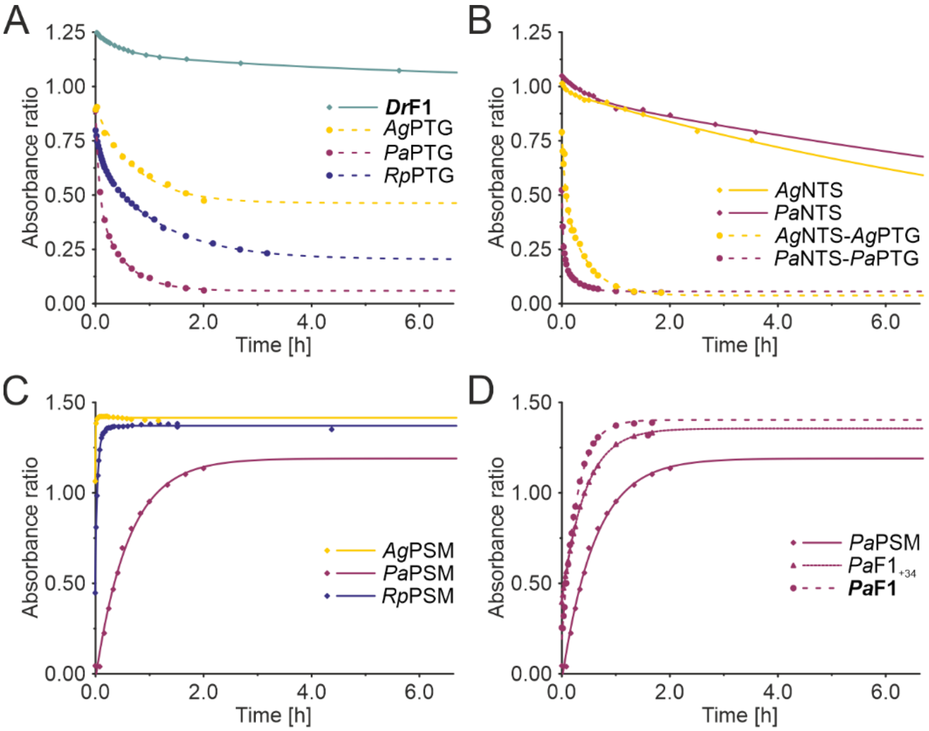

E

Sample	exp	$A_{max/min}$	τ_1 (min)	A_1 (%)	τ_2 (min)	A_2 (%)
***Dr*F1**	2	1.016	0.042	39	0.003	61
*Ag*PTG	1	0.464	0.023	100	-	-
*Pa*PTG	2	0.059	0.256	51	0.034	49
*Rp*PTG	2	0.204	0.154	23	0.014	77
*Ag*NTS	2	0.087	0.187	5	0.001	95
*Pa*NTS	2	0.001	0.039	9	0.001	91
*Ag*NTS-*Ag*PTG	2	0.038	0.274	39	0.039	61
*Pa*NTS-*Pa*PTG	2	0.056	0.660	65	0.075	35
*Ag*PSM	1	1.415	2.399	100	-	-
*Pa*PSM	1	1.190	0.027	100	-	-
*Rp*PSM	2	1.371	2.626	16	0.347	84
*Pa*F1$_{+34}$	1	1.356	0.039	100	-	-
***Pa*F1**	1	1.403	0.062	100	-	-

Figure S3: ***Dark reversion of DrF1-bathy chimaeras,*** *plotted as the ratio of absorbance at 750/700 nm.* ***(A)*** *DrF1 and PHY-tongue (PTG)-exchange variants (Fig. 3A–C).* ***(B)*** *N-terminal segment (NTS)-exchange variants and NTS+PTG-exchange variants (Fig. S4A–C).* ***(C)*** *Photosensory module (PSM)- and PSM-DrPTG-exchange variants (Fig. 3A,D; Fig. S4A,D). The recovery kinetics of AgPSM-DrPTG, PaPSM-DrPTG and RpPSM-DrPTG could not be evaluated; all three constructs appear locked in Pr (Fig. S4D, Fig. S5).* ***(D)*** *PaPSM and its linker variants PaF1$_{+34}$ and PaF1 (Fig. 5D).* ***(E)*** *Time constants (τ_n) and decay amplitudes (A_n) of the reversions. "exp" denotes the number of exponentials, and $A_{max/min}$ denotes the maximum absorption ratio at t = 0 min. The data was fitted using the formula* $\frac{A_{750}}{A_{700}}(t) = A_1 e^{-\frac{t}{\tau_1}} + A_2 e^{-\frac{t}{\tau_2}}$.

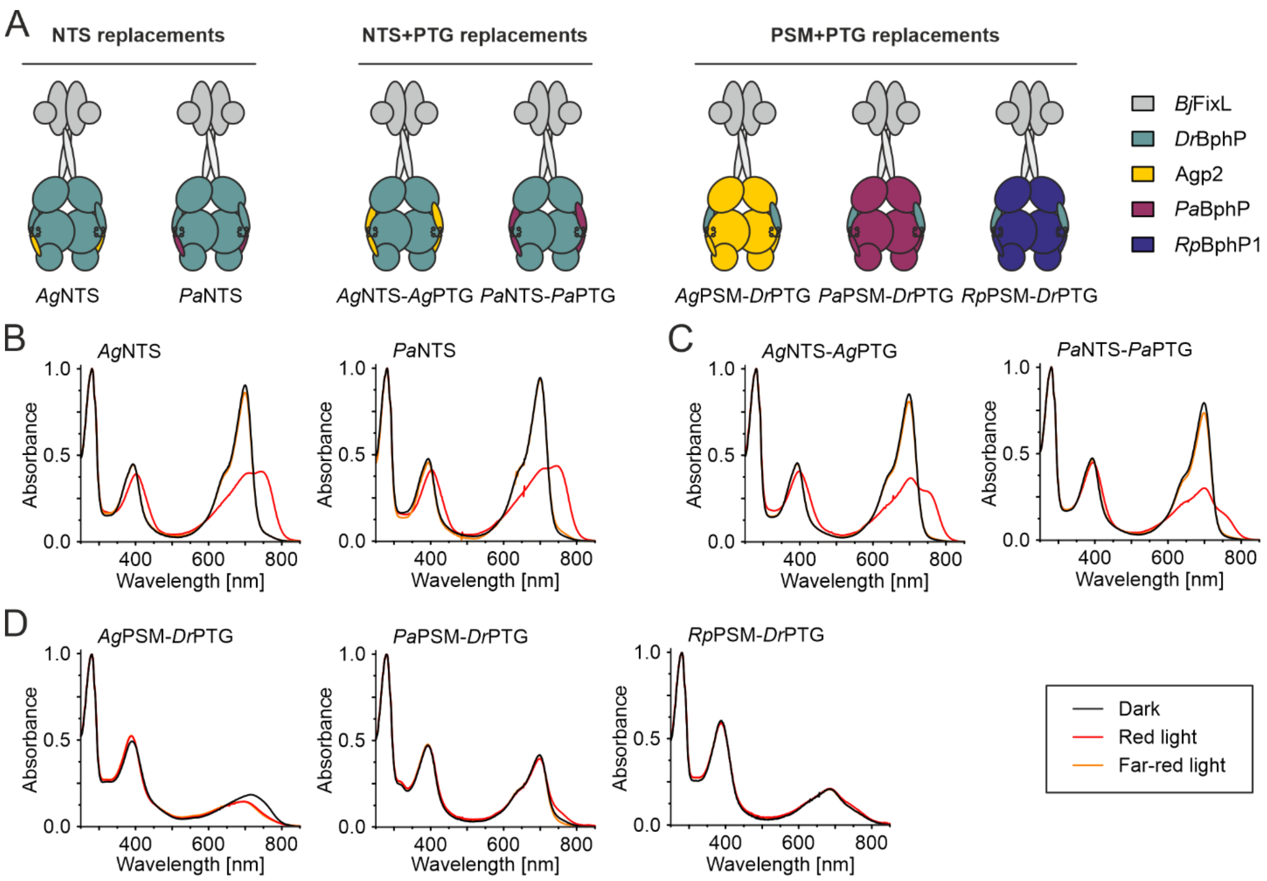

Figure S4: ***Additional DrF1-bathy chimeras and their absorption spectroscopy.*** ***(A)*** *Schematic presentation of additional DrF1 chimeras containing structural elements from three different bathy BphPs. Origins of the derived domain exchanges are colour-coded as indicated, see Table S2 for domain boundary definitions.* ***(B)*** *Absorption spectra of DrF1 chimaeras where the N-terminal segment (NTS) was exchanged for the corresponding bathy sequence.* ***(C)*** *Absorption spectra of DrF1 chimeras where both NTS and PHY-tongue (PTG) were exchanged for the corresponding bathy sequence. Low Pfr contribution in the red light illuminated state is likely a result of notably fast dark reversion (Fig. S3B)* ***(D)*** *Absorption spectra of bathy PSM constructs where the PTG was exchanged back to the DrBphP sequence. The resulting constructs undergo only minor shifts upon illumination with either red or far-red light. The Q-band intensities of these three chimaeras are also notably low. Since BV peak intensities are similar for all chimaeras after urea denaturation (Fig. S5), the reduced Q-band extinction coefficients cannot be assigned to incomplete or faulty BV loading. All spectra are normalised to* A_{280}.

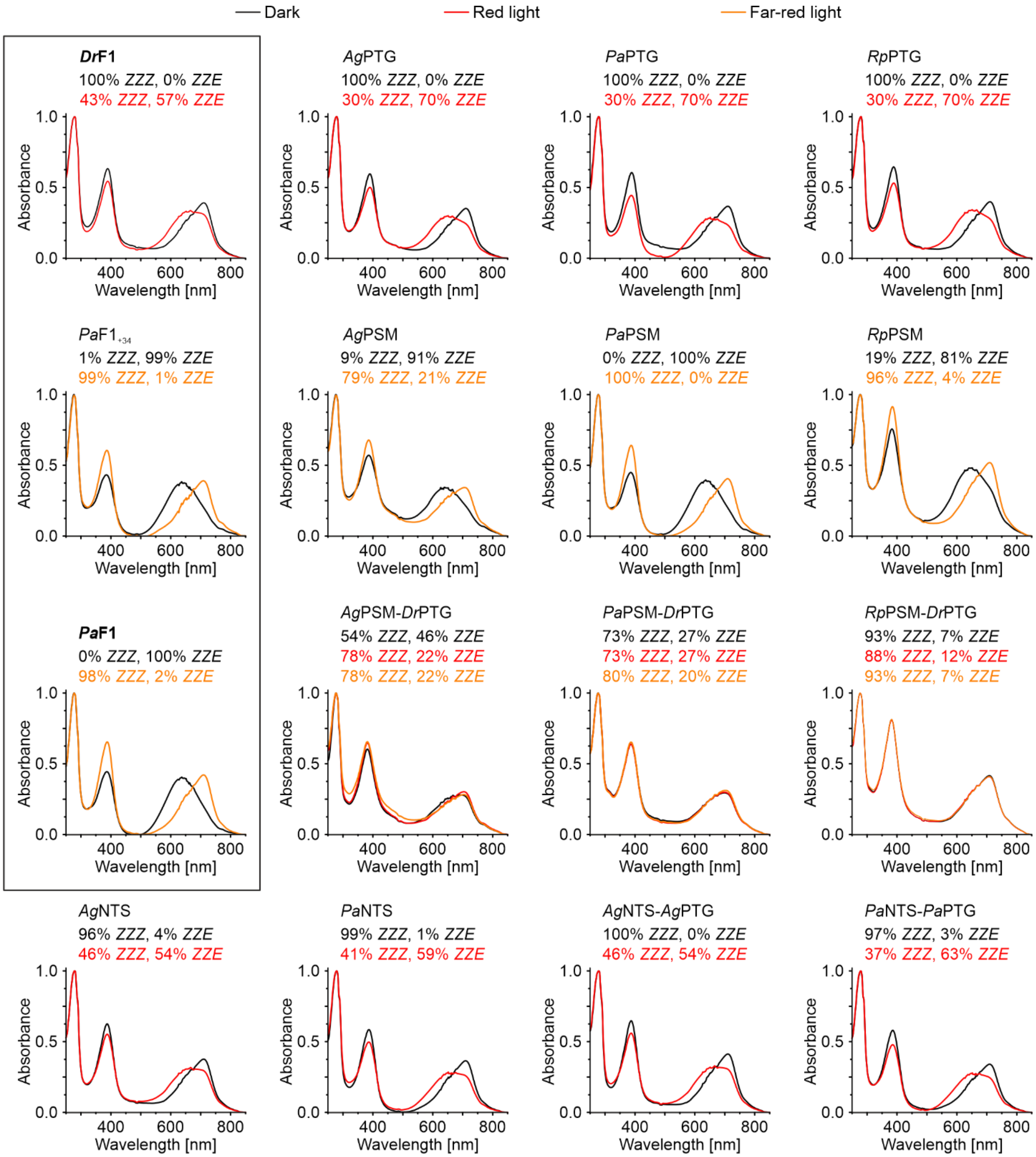

Figure S5: ***UV/Vis analysis of bathy DrF1 chimaeras denatured with urea*** *at pH 3.0 to assess biliverdin (BV) isomerisation. 0.4 mg* mL^{1} *protein were denatured after nonactinic / 3 min illuminated (662 / 782 nm) incubation and spectra were recoded using an Agilent Cary 8454 UVVisible spectrophotometer. Spectra are normalised to* A_{280} *= 1 and scattering effects were subtracted. Percentages of BV in ZZZ and ZZE were calculated based on the assumption that the ZZE content in the Pr/Pfr*

equilibrium reached after red light illumination equals 70% in DrPSM [5]*, as previously described* [6]*. AgPSM-DrPTG, PaPSM-DrPTG and RpPSM-DrPTG are locked in the Pr state – in contrast to all other chimaeras that undergo Z/E isomerisation. Thus, DrPTG appears to lock the chromophore in a Pr like conformation within the otherwise bathy PSM environment, eliminating the canonical light response.*

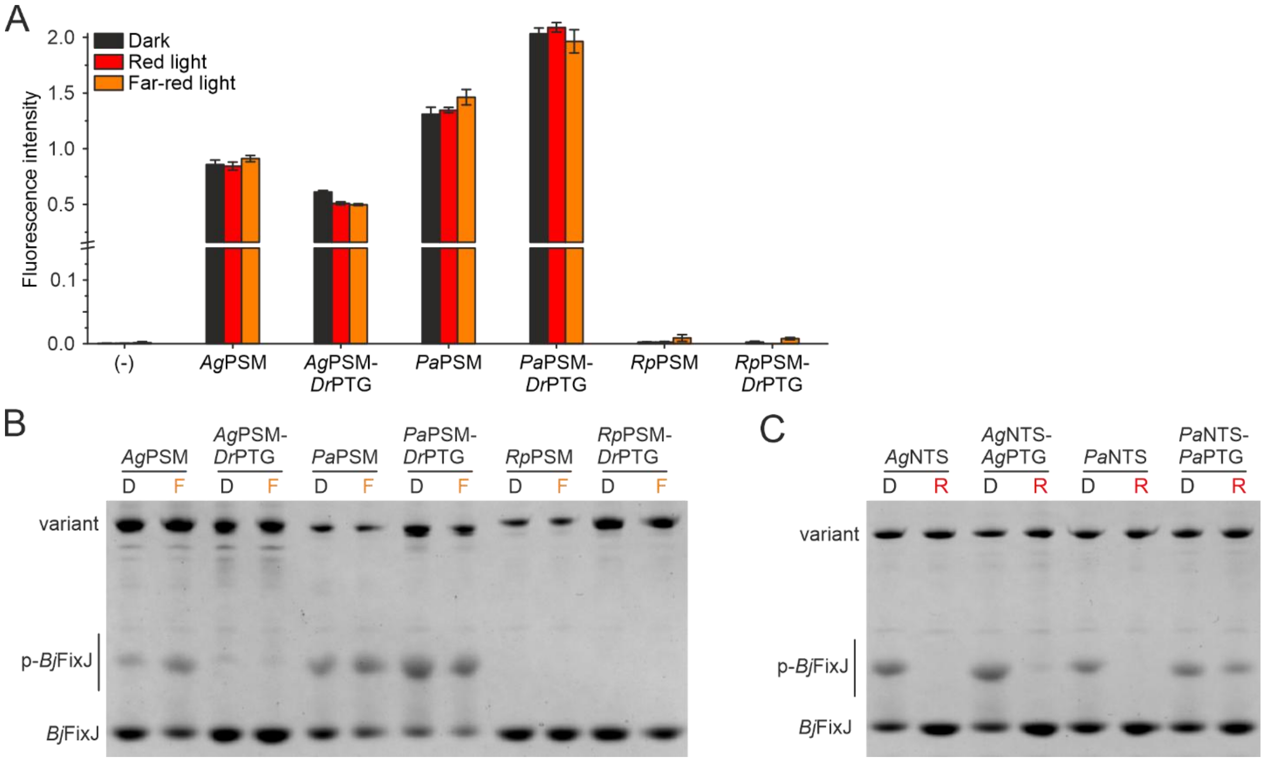

Figure S6: ***Activity assays of additional DrF1-bathy chimeras. (A)*** *Bacterial activity assay of PSM-DrPTG exchanges (Fig. S4A,D). The PSM exchanges from Figure 4 are also shown for comparison. pREDusk with the DsRed gene replaced by a multiple cloning site (MCS) functions as negative control (-).* ***(B)*** *Phos-tag activity assay PSM-DrPTG exchanges in dark (D) or in far-ref light (F). The PSM exchanges from Figure 4 are shown for comparison.* ***(C)*** *Phos-tag activity assay of N-terminal segment (NTS) exchanges (Fig. S4A,C), with and without corresponding PTG exchanges, in dark (D) or in red light (R). Samples were pre-illuminated with inducing light, mixed with ATP and incubated for 20 min under red (657 nm) / far-red (780 nm) light before separation on a polyacrylamide gel containing Phos-tag acrylamide, as previously described* [7].

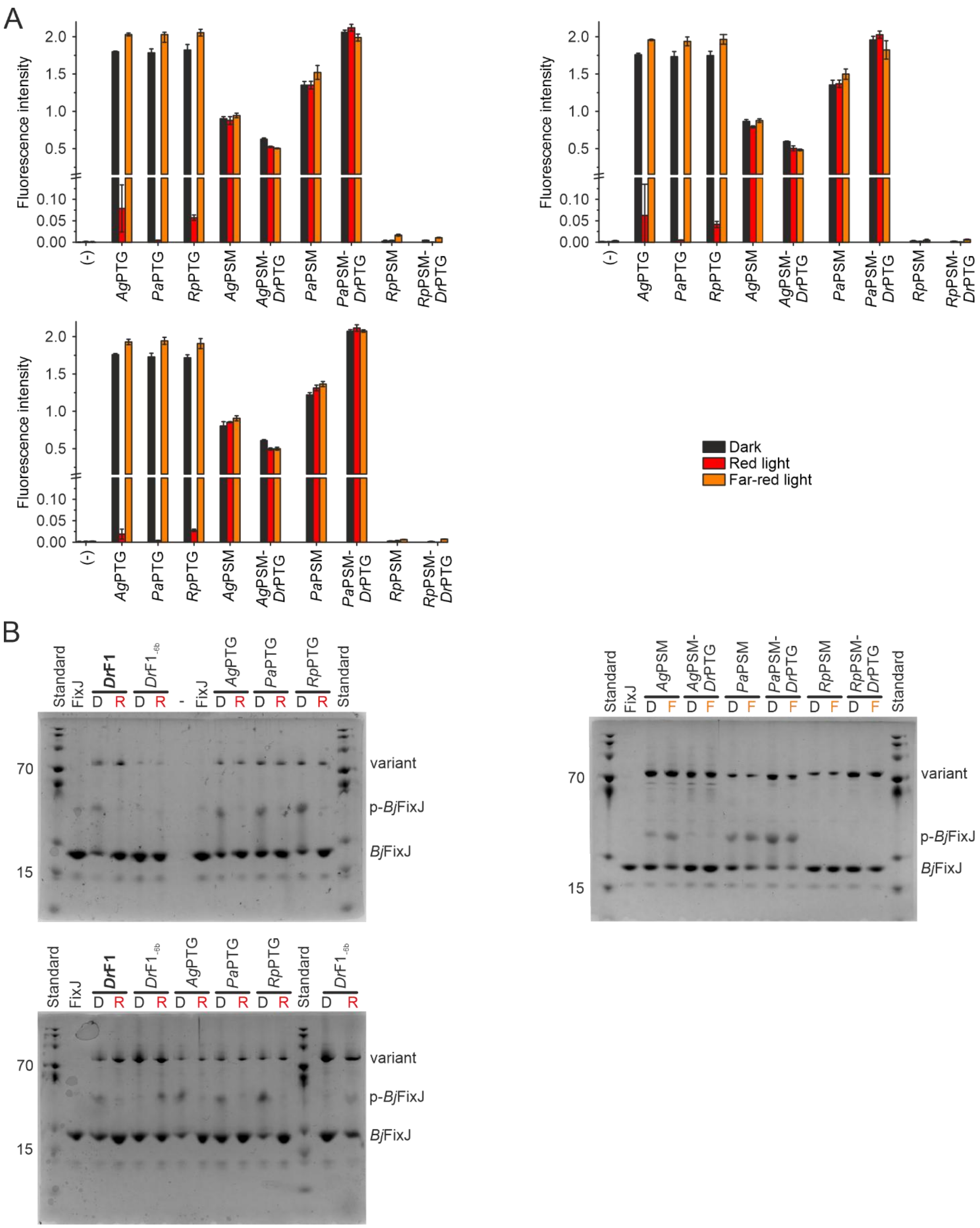

Figure S7: ***In vivo & in vitro HK activity assays. (A)*** *Individual biological replicates of measurements depicted in Figure 4A and Figure S6. DsRed fluorescence (normalised to DrF1, dark) corresponds to histidine phosphorylation,*

phosphotransfer to BjFixJ, and subsequent target gene expression. Individual biological repeats shown as mean ± SD of three technical repeats. pREDusk with the DsRed gene replaced by a multiple cloning site (MCS) functions as negative control (-). ***(B)*** *Full gels as depicted in Figure 4B. Samples were pre-illuminated with inducing light, mixed with ATP and incubated for 20 min under red (657 nm) / far-red (780 nm) light before separation on a polyacrylamide gel containing Phos-tag acrylamide, as previously described* [7]*. PageRuler Plus Prestained Protein Ladder for reference (kDa) – BphP samples have ~80 kDa mass, BjFixJ has ~23 kDa.*

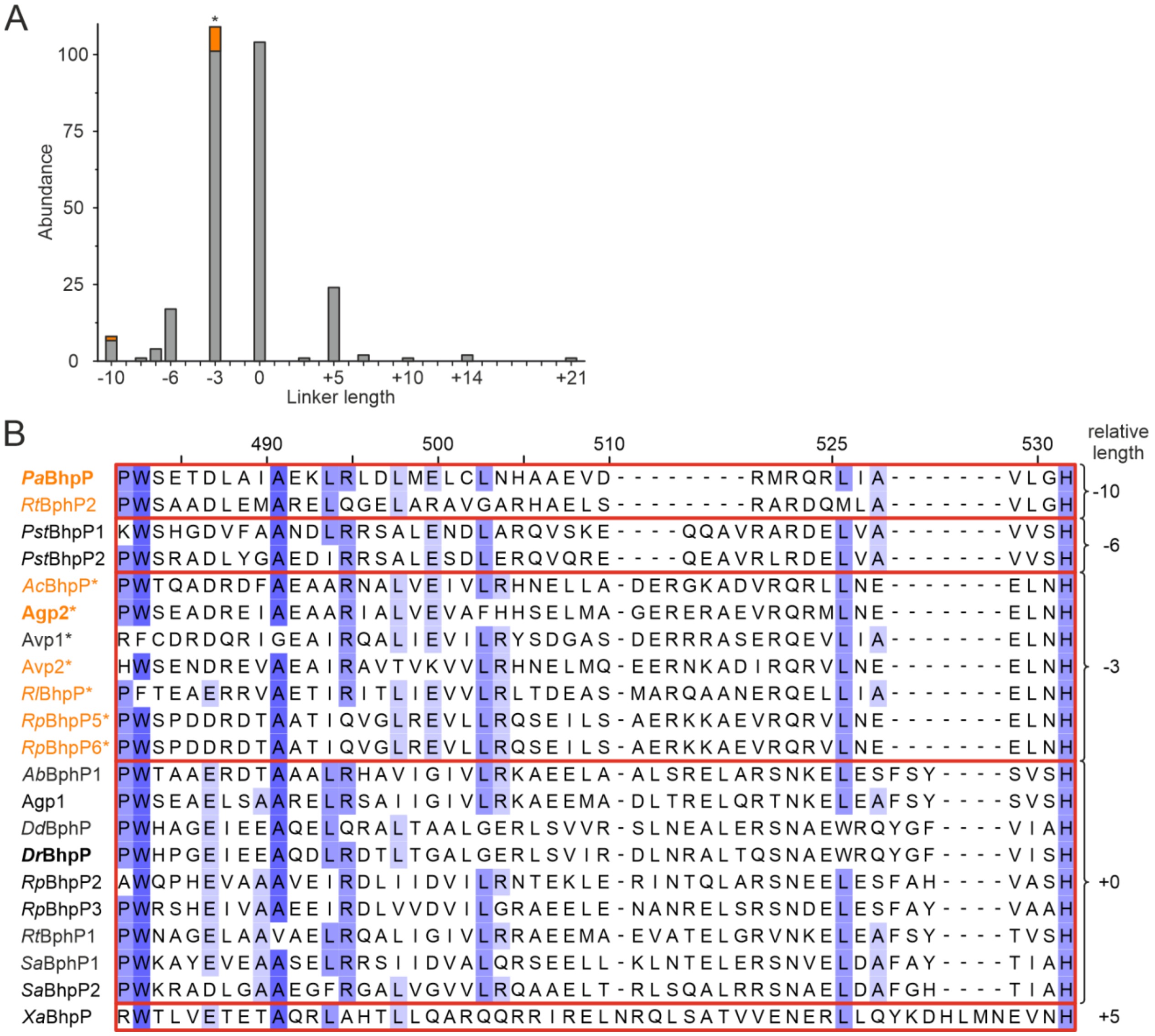

Figure S8: ***Linker lengths across BphPs. (A)*** *Absolute abundance of linker lengths in a total of 275 published BphPs with a HK or HWE-HK OPM, linker counted from the conserved PW motif (PRXSF+13, DrBphP P482) to the catalytic His residue (DrBphP H532) and given relative to DrBphP (51 residues). 107 are HWE-HKs (*), 106 of which have a linker length of 48 residues. Known bathy BphPs are represented in orange.* ***(B)*** *Alignment coloured according to Jalview sequence ID (hues of blue correspond to ≥ 84%, ≥ 68%, ≥ 40%, < 40% residue conservation), numbering corresponds to DrBphP. 404 BphP sequences were aligned with ClustalO default settings* [1] *(full alignment available as supporting file, "Supp_sequences_aligned.phy") and manually curated. HWE-HKs are marked with *, bathy BphPs are represented in orange.*

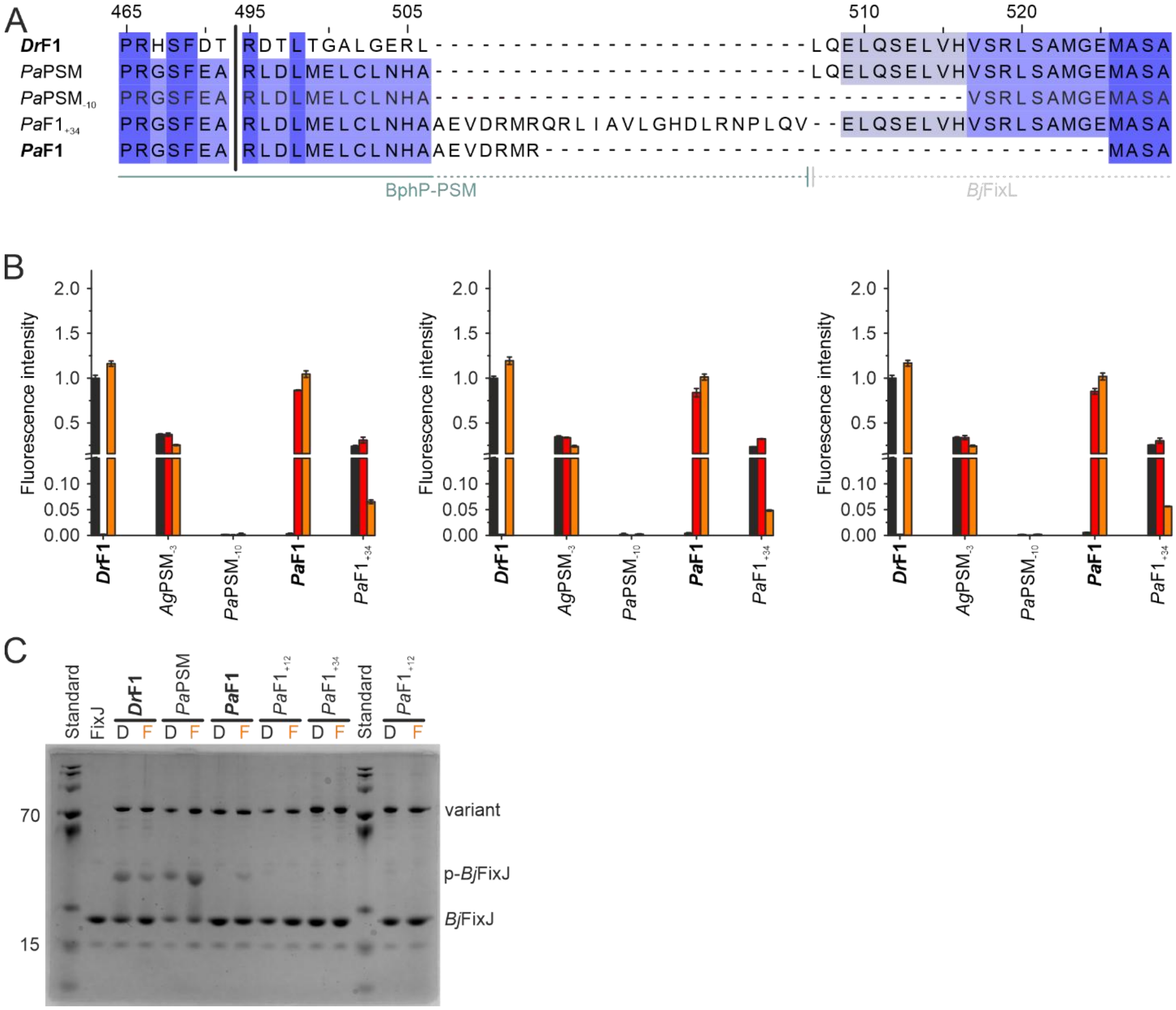

Figure S9: ***PaF1 activity in vitro and in vivo. (A)*** *Linker length comparison of AgPSM and PaPSM linker deletions. Coloured according to Jalview sequence ID (hues of blue correspond to ≥ 84%, ≥ 68%, ≥ 40%, < 40% residue conservation), numbering corresponds to DrBphP.* ***(B)*** *Individual biological replicates of measurements depicted in Figure 5B. DsRed fluorescence (normalised to DrF1, dark) corresponds to histidine phosphorylation, phosphotransfer to BjFixJ, and subsequent target gene expression. Individual biological repeats shown as mean ± SD of three technical repeats. pREDusk with the DsRed gene replaced by a multiple cloning site (MCS) functions as negative control (-).* ***(C)*** *Full gel as depicted in Figure 5C. Samples were pre-illuminated with inducing light, mixed with ATP and incubated for 20 min under red (657 nm) / far-red (780 nm) light before separation on a polyacrylamide gel containing Phos-tag acrylamide, as previously described* [7].

PageRuler Plus Prestained Protein Ladder for reference (kDa) – BphP samples have ~80 kDa mass, BjFixJ has ~23 kDa.

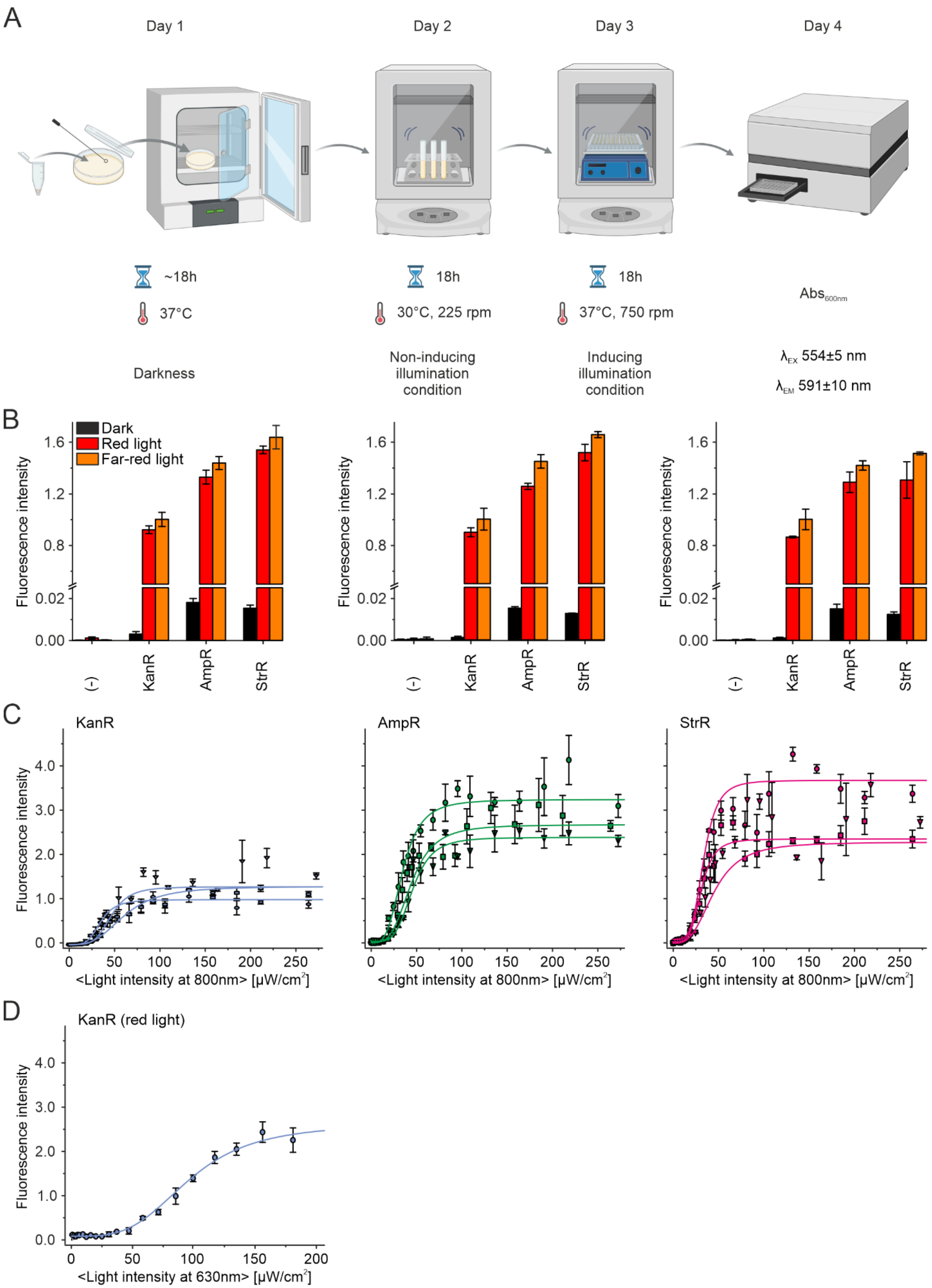
A
Day 1
Day 2
Day 3
Day 4
~18h
37°C
18h
30°C, 225 rpm
18h
37°C, 750 rpm
Abs600nm
Darkness
Non-inducing illumination condition
Inducing illumination condition
λEX 554±5 nm
λEM 591±10 nm
B
Dark
Red light
Far-red light
Fluorescence intensity
(-)
KanR
AmpR
StrR
C
KanR
AmpR
StrR
<Light intensity at 800nm> [µW/cm2]
D
KanR (red light)
<Light intensity at 630nm> [µW/cm2]

Figure S10: ***Assessment of pFREDusk as an optogenetic tool. (A)*** *Schematic representation of in vivo kinase activity workflow. Depiction of the 4-day protocol, adjusted from Multamäki et al.* [8]*. The figure was created with BioRender.com.* ***(B)*** *Individual biological replicates of in vivo HK activity measurements depicted in Figure 6B. DsRed fluorescence corresponds to histidine phosphorylation, phosphotransfer to BjFixJ, and subsequent target gene expression. Individual biological repeats shown as mean ± SD of three technical repeats, normalised to KanR in far-red. pREDusk with the DsRed gene replaced by a multiple cloning site (MCS) functions as negative control (-).* ***(C)*** *Individual replicates of light sensitivity measurements depicted in Figure 6C. DsRed production in bacteria harbouring pFREDusk with KanR (blue), AmpR (green) and StrR (pink) in response to varied far-red light (800 nm) intensities. Fluorescence intensity normalised to far-red light illuminated KanR endpoint. Light intensities are averaged over the duty cycle as marked by angled brackets.* ***(D)*** *DsRed production in bacteria harbouring pFREDusk with KanR in response to varied red light (630 nm) intensities, fluorescence intensity normalised to far-red light illuminated KanR endpoint.*

Table S1: ***Characterised BphPs*** *with known spectral properties as aligned in Figure 2 and Figure S8. Identifier as used in literature, parent organism, NCBI protein accession number and OPM.*

**ID**	**Species**	**NCBI**	**OPM**
*Ab*BphP1	*Azospirillum brasilense*	WP_040138077	HK
*Ac*BphP ORS 571	*Azorhizobium caulinodans*	WP_012169446	HWE-HK
Agp1	*Agrobacterium tumefaciens* F2	EGP56260	HK
Agp2	*Agrobacterium tumefaciens*	WP_174008917	HWE-HK
Avp1	*Allorhizobium ampelinum*	WP_012650515	HWE-HK
Avp2	*Allorhizobium ampelinum*	WP_015916920	HWE-HK
*Br*BphP1	*Bradyrhizobium sp.* ORS 278	WP_011924736	PAC
*Dd*BphP	*Deinococcus deserti*	WP_012695249	GHKL
*Dr*BphP	*Deinococcus radiodurans*	WP_010889310	HK
*Is*PadC	*Idiomarina sp.* A28L	WP_007419415	DGC
*Ma*PadC	*Marinimicrobium agarilyticum*	WP_027329460	DGC
*Mp*PadC	*Marinobacter persicus*	WP_091706258	DGC
*Pa*BphP	*Pseudomonas aeruginosa*	WP_034073960	HK
*Pst*bphP1	*Pseudomonas syringae* pv. *tomato*	KPY93576	HK
*Pst*bphP2	*Pseudomonas syringae* pv. *tomato*	WP_011104130	HK
*Rl*BphP	*Rhizobium leguminosarum*	WP_184697183	HWE-HK
*Rp*BphP1	*Rhodopseudomonas palustris*	WP_119846077	PAS/PAC
*Rp*BphP2	*Rhodopseudomonas palustris*	WP_011158562	HK
*Rp*BphP3	*Rhodopseudomonas palustris*	WP_011158563	HK
*Rp*BphP5	*Rhodopseudomonas palustris*	WP_119019282	HWE-HK
*Rp*BphP6	*Rhodopseudomonas palustris*	WP_011156523	HWE-HK
*Rs*BphG1, *Rhs*PadC-EAL	*Rhodobacter sp.* JA431	WP_097082434	DGC-EAL
*Rt*BphP1	*Ramlibacter tataouinensis* TTB310	AEG93643	HK
*Rt*BphP2	*Ramlibacter tataouinensis* TTB310	WP_013902229	HK
*Sa*BphP1	*Stigmatella aurantiaca*	WP_002612494	HK
*Sa*BphP2	*Stigmatella aurantiaca*	WP_002609371	HK
*Ts*PadC	*Thioalkalivibrio sp.* ALMg3	WP_026331574	DGC
*Xa*BphP	*Xanthobacter autotrophicus* Py2	ABS67686	HK
*Xcc*BphP	*Xanthomonas campestris*	WP_011270102	PAS

Table S2: ***Residue numbers of fusion points for bathy DrF1 chimaeras introduced in this study.*** *PSM = photosensory module; NTS = N-terminal segment; PTG = PHY-tongue; OPM = output module. PaPSM ext. was the template construct for PATCHY PCR.*

<table>
<tr><th>Construct</th><th>PSM</th><th>NTS/PTG</th><th>OPM</th></tr>
<tr><td>AgPSM</td><td>Agp2 4–500</td><td rowspan="3">—</td><td rowspan="3">BjFixL 266–505</td></tr>
<tr><td>PaPSM</td><td>PaBphP 1–497</td></tr>
<tr><td>RpPSM</td><td>RpBphP1 1–506</td></tr>
<tr><td>AgPTG</td><td rowspan="3">DrBphP 1–506</td><td>Agp2 435–471</td><td rowspan="3">BjFixL 266–505</td></tr>
<tr><td>PaPTG</td><td>PaBphP 432–468</td></tr>
<tr><td>RpPTG</td><td>RpBphP1 441–477</td></tr>
<tr><td>AgPSM-DrPTG</td><td>Agp2 4–500</td><td rowspan="3">DrBphP 446–477</td><td rowspan="3">BjFixL 266–505</td></tr>
<tr><td>PaPSM-DrPTG</td><td>PaBphP 1–498</td></tr>
<tr><td>RpPSM-DrPTG</td><td>RpBphP1 1–506</td></tr>
<tr><td>AgNTS</td><td rowspan="4">DrBphP 1–506</td><td>Agp2 2–11</td><td rowspan="4">BjFixL 266–505</td></tr>
<tr><td>PaNTS</td><td>PaBphP 2–18</td></tr>
<tr><td>AgNTS-AgPTG</td><td>Agp2 2–11, 435–471</td></tr>
<tr><td>PaNTS-PaPTG</td><td>PaBphP 2–18, 432–468</td></tr>
<tr><td>AgPSM$_{-3}$</td><td>Agp2 4–500</td><td rowspan="2">—</td><td>BjFixL 269–505</td></tr>
<tr><td>PaPSM$_{-10}$</td><td>PaBphP 1–497</td><td>BjFixL 276–505</td></tr>
<tr><td>PaPSM ext.</td><td>PaBphP 1–523</td><td>—</td><td>BjFixL 262–505</td></tr>
<tr><td>PaF1</td><td>PaBphP 1–504</td><td>—</td><td>BjFixL 285–505</td></tr>
<tr><td>PaF1$_{+34}$</td><td>PaBphP 1–521</td><td>—</td><td>BjFixL 268–505</td></tr>
</table>

Table S3: ***Genetic materials used in this study.*** *Antibiotic resistances: Kan = kanamycin; Amp = ampicillin; Str = streptomycin.*

**Construct**	**Parent plasmid**	**Insert**	**AntibioticR**	**Reference**
pREDusk	pREDusk-*Ds*Red	*Dr*F1	Kan	[8]
—	pREDusk-*Ds*Red	*Ag*PSM	Kan	—
—	pREDusk-*Ds*Red	*Pa*PSM	Kan	—
—	pREDusk-*Ds*Red	*Rp*PSM	Kan	—
—	pREDusk-*Ds*Red	*Ag*PTG	Kan	—
—	pREDusk-*Ds*Red	*Pa*PTG	Kan	—
—	pREDusk-*Ds*Red	*Rp*PTG	Kan	—
—	pREDusk-*Ds*Red	*Ag*PSM*Dr*PTG	Kan	—
—	pREDusk-*Ds*Red	*Pa*PSM*Dr*PTG	Kan	—
—	pREDusk-*Ds*Red	*Rp*PSM*Dr*PTG	Kan	—
—	pREDusk-*Ds*Red	*Ag*PSM$_{-3}$	Kan	—
—	pREDusk-*Ds*Red	*Pa*PSM$_{-10}$	Kan	—
pFREDusk	pREDusk-*Ds*Red	*Pa*F1	Kan	—
pFREDusk	pREDusk-*Ds*Red	*Pa*F1	Strp	—
pFREDusk	pREDusk-*Ds*Red	*Pa*F1	Amp	—
—	pREDusk-*Ds*Red	*Pa*F1$_{+34}$	Kan	—
	pET21b(+)	*Dr*F1	Amp	[8]
	pET21b(+)	*Ag*PSM	Amp	—
	pET21b(+)	*Pa*PSM	Amp	—
	pET21b(+)	*Rp*PSM	Amp	—
	pET21b(+)	*Ag*PTG	Amp	—
	pET21b(+)	*Pa*PTG	Amp	—
	pET21b(+)	*Rp*PTG	Amp	—
	pET21b(+)	*Ag*PSM-*Dr*PTG	Amp	—
	pET21b(+)	*Pa*PSM-*Dr*PTG	Amp	—
	pET21b(+)	*Rp*PSM-*Dr*PTG	Amp	—
	pET21b(+)	*Ag*NTS	Amp	—
	pET21b(+)	*Ag*NTS-*Ag*PTG	Amp	—
	pET21b(+)	*Pa*NTS	Amp	—
	pET21b(+)	*Pa*NTS-*Pa*PTG	Amp	—
	pET21b(+)	*Pa*F1	Amp	—
	pET21b(+)	*Pa*F1$_{+34}$	Amp	—

Table S4: ***Reverse primers used for PATCHY creation of PaPSM linker library.*** *Forward primers are listed in Meier et al.* [9].

Primer	Sequence	Primer	Sequence
Pa_3742_rev	CTGCAGCGGATTACGC	Pa_3703_rev	CAGACGCTGACGCATAC
Pa_3739_rev	CAGCGGATTACGCAGATC	Pa_3700_rev	ACGCTGACGCATACGATC
Pa_3736_rev	CGGATTACGCAGATCATG	Pa_3697_rev	CTGACGCATACGATCAAC
Pa_3733_rev	ATTACGCAGATCATGACC	Pa_3694_rev	ACGCATACGATCAACTTCC
Pa_3730_rev	ACGCAGATCATGACCC	Pa_3691_rev	CATACGATCAACTTCCGC
Pa_3727_rev	CAGATCATGACCCAGAACTG	Pa_3688_rev	ACGATCAACTTCCGCC
Pa_3724_rev	ATCATGACCCAGAACTGC	Pa_3685_rev	ATCAACTTCCGCCGCATG
Pa_3721_rev	ATGACCCAGAACTGCAATC	Pa_3682_rev	AACTTCCGCCGCATGG
Pa_3718_rev	ACCCAGAACTGCAATCAG	Pa_3679_rev	TTCCGCCGCATGGTTG
Pa_3715_rev	CAGAACTGCAATCAGACG	Pa_3676_rev	CGCCGCATGGTTGAGG
Pa_3712_rev	AACTGCAATCAGACGC	Pa_3673_rev	CGCATGGTTGAGGCAC
Pa_3709_rev	TGCAATCAGACGCTGAC	Pa_3670_rev	ATGGTTGAGGCACAGTTC
Pa_3706_rev	AATCAGACGCTGACGC	Pa_3667_rev	GTTGAGGCACAGTTCCATC

SUPPLEMENTARY REFERENCES

1 Sievers, F., Wilm, A., Dineen, D., Gibson, T. J., Karplus, K., Li, W., et al. (2011) Fast, scalable generation of high-quality protein multiple sequence alignments using Clustal Omega. *Mol Syst Biol.* 7, 539

2 Felsenstein, J. (2005) PHYLIP (Phylogeny Inference Package) version 3.6, Distributed by the author. Department of Genome Sciences, University of Washington, Seattle

3 Rambaut, A. (2018) FigTree v1.4.4.

4 Wu, X. and Bartel, D. P. (2017) *k*pLogo: positional *k*-mer analysis reveals hidden specificity in biological sequences. *Nucleic Acids Res.* 45, W534–W538

5 Takala, H., Lehtivuori, H. K., Berntsson, O., Hughes, A., Nanekar, R., Niebling, S., et al. (2018) On the (un)coupling of the chromophore, tongue interactions, and overall conformation in a bacterial phytochrome. *J Biol Chem.* 293, 8161–8172

6 Lenngren, N., Edlund, P., Takala, H., Stucki-Buchli, B., Rumfeldt, J., Peshev, I., et al. (2018) Coordination of the biliverdin *D*-ring in bacteriophytochromes. *Phys Chem Chem Phys* **20**, 18216–18225

7 Multamäki, E., Nanekar, R., Morozov, D., Lievonen, T., Golonka, D., Wahlgren, W. Y., et al. (2021) Comparative analysis of two paradigm bacteriophytochromes reveals opposite functionalities in two-component signaling. *Nat Commun.* 12, 4394

8 Multamäki, E., García de Fuentes, A., Sieryi, O., Bykov, A., Gerken, U., Ranzani, A. T., et al. (2022) Optogenetic Control of Bacterial Expression by Red Light. *ACS Synth Biol.* 11, 3354–3367
